# Therapeutic Potential of Non-Psychotropic Cannabidiol in Ischemic Stroke

**DOI:** 10.3390/ph3072197

**Published:** 2010-07-08

**Authors:** Kazuhide Hayakawa, Kenichi Mishima, Michihiro Fujiwara

**Affiliations:** Department of Neuropharmacology, Faculty of Pharmaceutical Sciences, Fukuoka University, 8-19-1 Nanakuma, Jonan-ku, Fukuoka, Japan; E-Mails: kenichi@fukuoka-u.ac.jp (K.M.); mfuji@fukuoka-u.ac.jp (M.F.)

**Keywords:** cannabinoids, cannabidiol, ischemic stroke, neuroprotective effect

## Abstract

Cannabis contains the psychoactive component delta^9^-tetrahydrocannabinol (delta^9^-THC), and the non-psychoactive components cannabidiol (CBD), cannabinol, and cannabigerol. It is well-known that delta^9^-THC and other cannabinoid CB_1_ receptor agonists are neuroprotective during global and focal ischemic injury. Additionally, delta^9^-THC also mediates psychological effects through the activation of the CB_1_ receptor in the central nervous system. In addition to the CB_1_ receptor agonists, cannabis also contains therapeutically active components which are CB_1_ receptor independent. Of the CB_1_ receptor-independent cannabis, the most important is CBD. In the past five years, an increasing number of publications have focused on the discovery of the anti-inflammatory, anti-oxidant, and neuroprotective effects of CBD. In particular, CBD exerts positive pharmacological effects in ischemic stroke and other chronic diseases, including Parkinson’s disease, Alzheimer’s disease, and rheumatoid arthritis. The cerebroprotective action of CBD is CB_1_ receptor-independent, long-lasting, and has potent anti-oxidant activity. Importantly, CBD use does not lead to tolerance. In this review, we will discuss the therapeutic possibility of CBD as a cerebroprotective agent, highlighting recent pharmacological advances, novel mechanisms, and therapeutic time window of CBD in ischemic stroke.

## 1. Introduction

**Figure 1:**
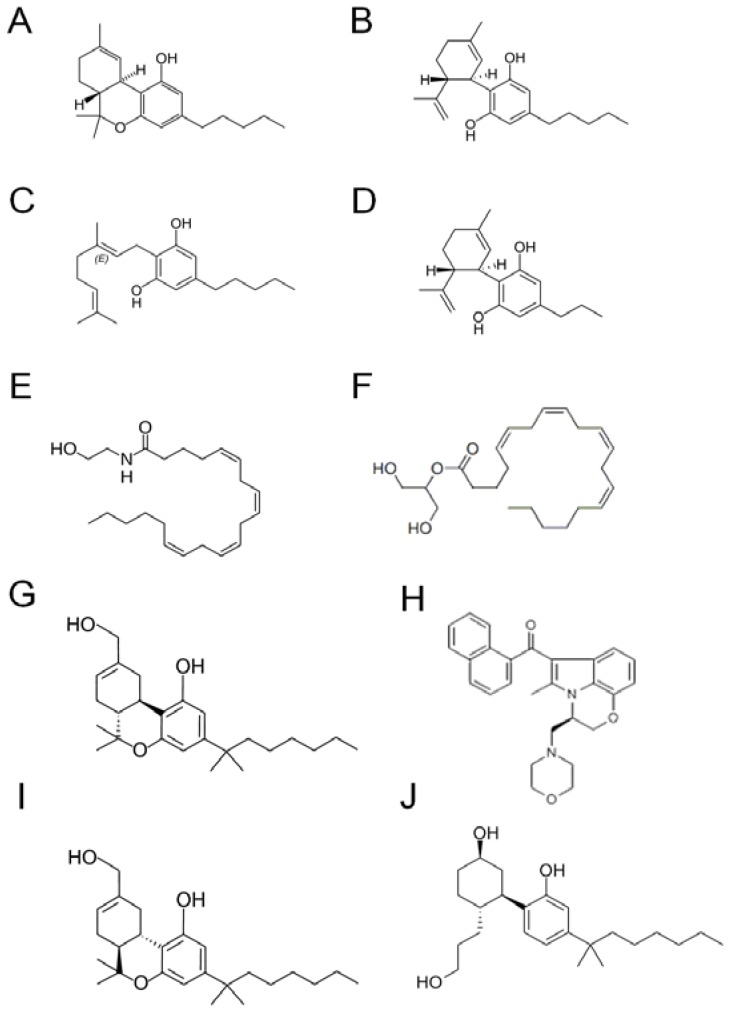
Cannabinoid structures. (A) Delta^9^-tetrahydrocannabinol (delta^9^-THC); (B) cannabidiol (CBD); (C) cannabigerol (CBG); (D) cannabidivarin (CBDV); (E) anandamide (AEA); (F) 2-arachidonyl glycerol (2-AG); (G) HU210; (H) WIN55, 212-2; (I) HU211; (J) CP55940; (A)–(D) natural cannabinoids; (E) and (F) endocannabinoids; (G)–(J) synthetic cannabinoids.

Cannabis contains over 60 different terpeno-phenol compounds that have been identified so far but the role and importance of many of these has yet to be fully understood. Select structures are shown in Figure 1. Delta^9^-tetrahydrocannabinol (delta^9^-THC) is the most psychoactive component that was isolated in 1964 by Gaoni and Mechoulam at the Weizmann Institute in Rehovot (Israel). It has been demonstrated to produce hypothermia, learning and memory impairment, impairment of the prepulse inhibition of the startle reflex, catalepsy-like immobilisation, aggressive behaviour, analgesia, hypoactivity and enhancement of preference for high fat diet [1,2,3,4,5,6,84,85,86,87,88]. These effects are at least partly caused by binding to cannabinoid receptor type 1 (CB_1_) within the brain. So far, two types of cannabinoid receptors have been identified: type1 (CB_1_ receptor) and type2 (CB_2_ receptor). CB_1_ receptors are mainly expressed in the central and the peripheral nervous system. CB_2_ receptors are found in cells of the immune system, such as lymphocytes and neutrophils, as well as in resident inflammatory cells within the CNS [7,8,9]. 

On the other hand, cannabidiol (CBD), cannabigerol (CBG), cannabidivarin (CBDV) are known as non-psychoactive components of cannabis. These compounds have shown anti-inflammatory, immunosuppressive, analgesic, anxiolytic and anti-cancer effects [10]. In particular, much attention has been focused on CBD, which constitutes up to 3–4% of the cannabis extract. CBD was first isolated in 1940 by Adams and coworkers, but its structure and stereochemistry were only determined in 1963 by Mechoulam and Shvo. CBD has very low affinity (in the micromolar range) for the CB_1_ receptor as well as for the CB_2_ receptor and was found to be an anticonvulsant in animal models of epilepsy and in humans with epilepsy. Moreover, CBD has anti-spasmodic, anxiolytic, antinausea and anti-rheumatoid arthritis properties [11]. CBD has also been shown to be protective against *N*-methyl-D-aspartate and beta-amyloid peptide toxicity [12], as well as global and focal ischemic injury [13,14]. More recently, CBD has been found to have diverse additional functions, such as the neuroprotective effect induced through directly interacting with mitochondria-dependent Ca^2+^ regulation [79], the enhancement of adenosine signaling by inhibiting its uptake [15], blocking new cannabinoid receptor GPR55 [16], and inhibition of high-mobility group box1 activity.

In this review we will discuss the recent development of “CBD pharmacology” in ischemic stroke, focusing on the therapeutic potential of non-psychoactive cannabinoid CBD highlighting recent pharmacological advances, new mechanisms of action, and the therapeutic time window for its use in ischemic stroke.

## 2. Pharmacology of CBD in Ischemic Stroke

### 2.1. Cannabinoid Receptors and Endocannabinoid System

Accumulating data now suggest that cannabinoid CB_1_ receptors contribute to neuroprotection through anti-excitotoxicity [14], the phosphatidylinositol-3 kinase/Akt pathway [17], and hypothermia [18]. We have reported that the neuroprotective and hypothermic effects of delta^9^-THC were related to CB_1_ receptors [89]. CBD has also been described as protective against global and focal ischemic injury [14,18]. Several groups have recently shown that CBD can interact with cannabinoid CB_1_ and CB_2_ receptors. Pertwee and Ross showed that CBD can antagonize the CB_1_ agonists such as WIN55,212 and CP55940 by acting at prejunctional sites that was unlikely to be cannabinoid CB_1_ or CB_2_ receptors [19]. Castillo and coworkers recently demonstrated that CBD implicated the neuroprotective effect mediated by CB_2_ and adenosine receptors in an *in vitro* model of newborn hypoxic-ischemic brain damage in mice [21]. In addition, Thomas and coworkers noted that CBD displayed unexpected high potency as an antagonist of CB_1_ and CB_2_ receptor agonists [20]. Interestingly, the neuroprotective effect of CBD showed a dose dependent bell shaped curve in mice subjected to middle cerebral artery occlusion (MCAO) [22]. Intraperitoneal injection of CBD 1 or 3 mg/kg but not 10 mg/kg during 4 h MCAO prevented cerebral infarction 24 hours after cerebral ischemia. Other groups have demonstrated that 5 mg/kg was the most effective CBD dose, and that there was a dose-dependent bell-shaped curve for CBD’s effects on the electroencephalographic flattening in gerbils subjected to cerebral ischemia [13]. CBD has been also reported to inhibit anandamide amidase [23] and the reuptake of anandamide [24], suggesting CBD may induce an increase of anandamide signaling within the ischemic brain. Anandamide and other CB_1_ receptor agonists are known to reduce the release of a variety of neurotransmitters including glutamate via CB_1_ receptor [25,26,27,28,29]. In our previous study, delta^9^-THC significant reduced the release of glutamate during MCAO, but CBD did not affect glutamate release [30], suggesting that CBD might have other mechanism or stimulate other receptors besides the cannabinoid receptors. The mechanism of this biphasic effect of CBD is still unclear, but CBD may induce cerebroprotective effect through modulating endogenous cannabinoid system.

### 2.2. Serotonin 5-HT_1A_ Receptor-Dependent Mechanism

In 1974, an interactive study comparing CBD and THC in healthy volunteers demonstrated for the first time that CBD could act as an anxiolityc drug [31]. Experimentally, the anxiolytic properties of CBD have been demonstrated in different animal models such as the conditioned emotional response, the Vogel conflict test, and the elevated plus-maze [32,33]. Recently, it has been shown that CBD might exert anxiolytic effects by activating post-synaptic 5-HT_1A_ receptors [34]. 5-HT_1A_ receptors have been shown to play critical roles in the pathophysiology of depression, aggression, and anxiety. It appears to have a role in vasodilatation and neuroprotection [35,36,37,38,39,40]. We previously applied a new approach to the investigation of the neuroprotective mechanism of CBD by examining the effects of a CB_1_ receptor antagonist, a vanilloid-receptor (VR1) antagonist, and a 5-HT_1A_ receptor antagonist in a 4h MCA occlusion model in mice. Interestingly, the neuroprotective effect of CBD was inhibited by the 5-HT_1A_ receptor antagonist, WAY100135. Furthermore, the increased cerebral blood flow induced by CBD was also in part reduced by WAY100135. In contrast, the CB_1_ receptor antagonist and VR1 did not inhibit the effect of CBD [22]. Russo and co-workers demonstrated that CBD, but not delta^9^-THC, displaced the 5HT_1A_ agonist, [^3^H]-8-hydroxy-2-di-n-propylaminotetralin ([^3^H]-8-OH-DPAT) from the cloned human 5HT_1A_ receptor in a dose-dependent manner, and they have concluded that CBD was an agonist of the 5HT_1A_ receptor [41]. More recently, Magen and co-workers supported that CBD (5 mg/kg, i.p.) induced activation of 5-HT_1A_ receptors located in forebrain regions including the hippocampus, and improved cognitive and locomotor function which were impaired by bile-duct ligation [42]. Taken together, these data indicate that CBD may activate the 5-HT_1A_ receptor which leads to the improvement of cognitive and functional impairment after cerebral ischemia.

### 2.3. Potent Anti-Oxidant Mechanism

In 1998, Hampson and co-workers noted that the nonpsychoactive marijuana constituent CBD prevented both glutamate neurotoxicity and ROS-induced cell death with a stronger effect than either of the dietary antioxidants, α-tocopherol or ascorbate [43]. Surprisingly, CBD protected neurons with comparable efficacy to the potent antioxidant, butylated hydroxytoluene (BHT). In same research group, CBD and delta^9^-THC suppressed the oxidation potential measured by cyclic voltammetry and CBD was more effective than delta^9^-THC, suggesting that cannabidiol may be a neuroprotective antioxidant [14]. We have assessed both CBD and delta^9^-THC for antioxidant activity using the original 1,1-diphenyl-2-picryhydrazyl (DPPH) radical method described by Brand-Williams *et al*. [44]. Our results showed that CBD exhibited stronger antioxidative power (EC_50_ = 89.2 μM) than delta^9^-THC (EC_50_ = 464.2 μM) [45]. In an *in vivo* study, Hamelink and co-workers found that CBD protected against hippocampal-entorhinal-cortical neurodegeneration caused by ethanol exposure in rats. They showed that this effect of CBD attributed its anti-oxidative action [46]. These data suggest that CBD may be a very useful therapeutic agent for oxidative disorders after ischemic stroke.

### 2.4. Cerebroprotective Effect without the Development of Tolerance

Repeated treatment with delta^9^-THC and other CB_1_ receptor agonists result in the development of tolerance to its most acute behavioral and pharmacological effects [47,48,49,50]. Delta^9^-THC has been shown to lead to tolerance of hypoactivity, hypothermia, anti-nociception, catalepsy and pentobarbital-induced sleep prolongation [51,52,53]. In fact, 14 day-repeated treatment with delta^9^-THC (10 mg/kg) led to tolerance of the hypothermic and also neuroprotective effects. Additionally, the expression levels of CB_1_ receptor were down-regulated in mice with repeated doses of 10 mg/kg [45]. On the other hand, repeated treatment with CBD did not lead to development of tolerance in the cerebroprotective effect on infarction. CBD induced an increase in cerebral blood flow (CBF) even after 14 days of repeated treatment. Since these effects of CBD were in part inhibited by WAY100135, CBD may not be full 5-HT_1A_ agonist. It is known that, 8-OH-DPAT, a complete 5HT_1A_ agonist, induces tolerance [54], while a 5-HT_1A_ receptor partial agonist such as buspirone does not [55]. For that reason, the neuroprotective effect of CBD may be mediated through partial agonistic effect on the 5-HT_1A_ receptor.

### 2.5. Potent Anti-Inflammatory Effect

Emerging data now support the evidence of the anti-inflammatory action of CBD. For example, CBD suppressed interleukin-1 and inducible nitric oxide synthease induced by beta-amyloid [56]. CBD suppressed tumor necrosis factor α production in lipopolysaccharide-treated mice via A_2A_ adenosine receptor activation, and this effect was reversed with an A_2A_ adenosine-receptor antagonist, and abolished in A_2A_ receptor knockout mice [15]. In addition, CBD inhibited neutrophil migration induced by formyl-methionyl-leucyl-phenylalanine in CB_1_ and CB_2_ receptor independent pathways which are antagonized by the endogenous compound *N*-arachdonoyl-L-serine [57]. We have reported that CBD had a cerebroprotective action via a cannabinoid receptor-independent myeloperoxidase inhibiting mechanism in a 4h MCA occlusion model in mice [30]. In this model, the neuroprotective effect of delta^9^-THC was only evident with pre-ischemic treatment via inhibition of the release of glutamate in *in vivo* microdialysis-HPLC measurements and hypothermia in CB_1_ receptor dependent mechanism. On the other hand, CBD was cerebroprotective even when administered 6 hours after cerebral ischemia. In addition, we demonstrated that CBD, but not delta^9^-THC, inhibited myeloperoxidase activity in neutrophils and prevented the decrease in cerebral blood flow due to the failure of cerebral microcirculation after reperfusion. In a more recent study, we also found that the anti-inflammatory action of CBD was associated with inhibition of plasma high-mobility group box1 leaked from dying cells and released from damaged cells and/or monocytes/macrophages [58]. These results suggest that CBD may prevent post-ischemic injury progressively induced by ischemic stroke.

## 3. Therapeutic Time Window of CBD and Other Cannabinoids in Ischemic Stroke

Focal cerebral ischemia induces a complex series of mechanisms. The pattern of excitory amino acid efflux in different models of cerebral ischemia derives from the finding that a massive release of glutamate in the ischemic early phase is considered to play a major role in inducing ischemic and post-ischemic cell death [59]. In fact, antagonists of glutamate receptors reduce the ischemic penumbra [60,61], and inhibitors of glutamate release exhibit cerebroprotective activity against ischemia/ reperfusion-evoked injury [62].

**Figure 2:**
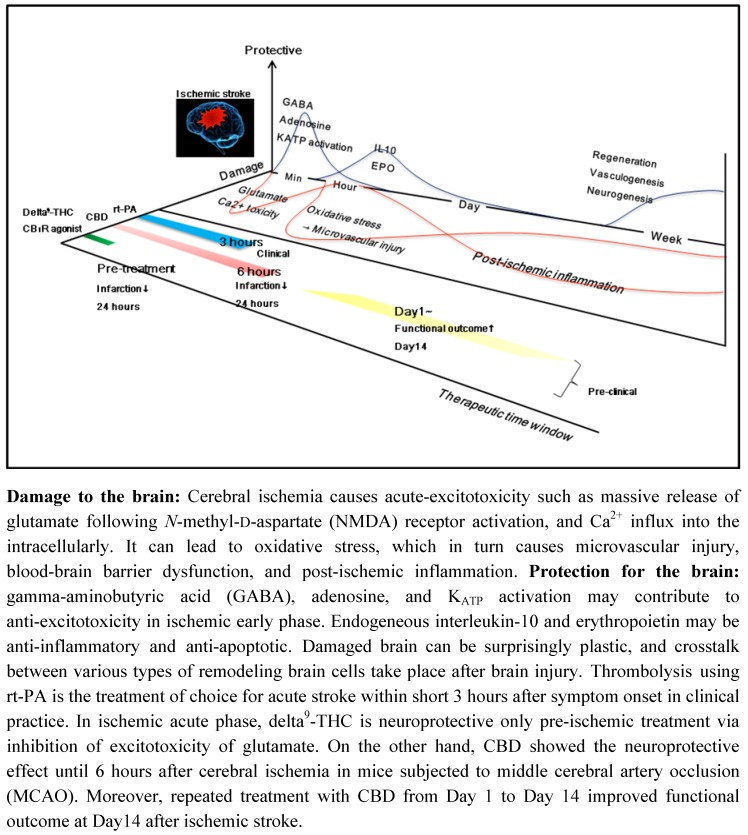
Therapeutic time window of CBD and delta^9^-THC in pre-clinical study.

Within seconds to minutes after an excitotoxic injury oxidative stress is evident and this in turn causes microvascular injury, blood-brain barrier dysfunction, and post-ischemic inflammation over the next 24 hours. Moreover, the initial ischemic event activates migroglia and astrocytes which react by secreting cytokines, chemokines, and matrix metalloproteases. These inflammatory mediators lead to an upregulation of intracellular adhesion molecule-1, E-selectin and P-selectin on endothelial cells, allowing blood derived inflammatory cells, mainly neutrophils, to infiltrate the ischemic brain area [63]. Damaged brain can be surprisingly plastic, and crosstalk between various types of remodeling brain cells take place after brain injury [64,65,66]. GABA, adenosine, and K_ATP_ activation may contribute to anti-excitotoxicity in the ischemic early phase. Endogeneous interleukin-10 and erythropoietin may lead to anti-inflammation and anti-apoptosis. In the late phase after cerebral ischemia, the generation of new blood vessels facilitates highly coupled neurorestorative processes including neurogenesis and synaptogenesis [67,68]. In fact, neurogenesis after stroke has been demonstrated in the adult human brain [69]. Thus, cerebral ischemia induces complex mechanisms progressively such as the processes of cell destruction and of cell protection/regeneration, which suggest that multifunctional molecule and compounds without interfering with beneficial endogenous mechanisms may become a candidate to prolong the therapeutic time window after ischemic stroke. In the search for therapies for stroke, to date over 1,026 drugs have been tested in animal models, of which 114 underwent further clinical evaluation [70]. Recombinant tissue plasminogen activator (rt-PA) remains the only agent shown to improve stroke outcome in clinical trials. However, patients are eligible for thrombolysis by using rt-PA only if they come to medical attention within a short time (3 hours) after symptom onset. This restriction is considered necessary due to the risk of hemorrhage and potential damage caused by ischemia/reperfusion injury. Research is now underway to expand the therapeutic time window for the use of thrombolytic therapy after cerebral ischemia.

### 3.1. CBD and Delta^9^-THC

We have previously reported that CBD (3 mg/kg) has a potent and long-lasting neuroprotective effect when administered both pre- and post-ischemia, whereas only pre-ischemic treatment with delta^9^-THC (10 mg/kg) reduced the infarction size when administered 24 hours after 4 h MCAo in mice. The neuroprotective effect of delta^9^-THC and other cannabinoids is related to the CB_1_ receptor-mediated inhibition of voltage-sensitive Ca^2+^ channels, which reduces Ca^2+^ influx, glutamate release, and excitotoxicity [12]. In fact, delta^9^-THC inhibited the massive release of glutamate, but this neuroprotective effect of delta-^9^THC was inhibited by the CB_1_ receptor antagonist SR141716 [45], which suggest that delta^9^-THC is neuroprotective as a pre-ischemic treatment via inhibition of glutamate excitotoxicity. Actually, treatment with delta^9^-THC immediately after reperfusion did not prevent cerebral infarction. On the other hand, CBD was neuroprotective even when administered 6 hours after cerebral ischemia (2 hours after reperfusion). In addition, CBD significantly inhibited the myeloperoxidase activity of neutrophils at 1 hour and 20 hours after reperfusion and suppressed the decrease in CBF due to the failure of cerebral microcirculation after reperfusion. In addition, CBD decreased the number of Iba1- and GFAP-positive cells and improved neurological score and motor coordination at 3 days after cerebral ischemia. We concluded that the therapeutic time window of CBD was 6 hours after onset of ischemia in early treatment. CBD had a potent and long-lasting neuro- protective effect and prevented progressive post-ischemic injury [58]. In a more recent study, we reported that repeated treatment with CBD from 1 day or 3 days after cerebral ischemia improved the functional deficits, such as neurological score and motor coordination, and survival rates. In addition, both groups did not increase the HMGB1 level in plasma, and decreased the number of Iba1 expressing HMGB1 positive cells and TUNEL positive cells, which suggest that CBD may be cerebroprotective not only during the early phase, but also during the chronic phase after ischemic stroke [71]. However, treatment with CBD from Day 5 did not improve the functional outcome Day 14 after cerebral ischemia. As described above, CBD has a potent anti-inflammatory effect, such as inhibition of glial activation. Activated microglia and reactive astrocytes have also a role of beneficial in ischemic brain. For example, microglia can produce neurotorophic factors such as brain-derived neurotorophic factor, insulin-like growth factor 1 [95]. And, reactive astrocytes can also release many growth factors such as nerve growth factor [96]. Taken together, a treatment with CBD in ischemic early phase may implicate the neuroprotective action through inhibition of acute inflammatory reaction, whereas ischemic delayed treatment with CBD may interfere with beneficial endogenous mechanisms. 

### 3.2. Synthetic CB_1_ Receptor Agonist, HU210 and R*(+)*-WIN55,212-2

Leker and co-workers investigated the therapeutic time window of the synthetic CB_1_ receptor agonist, HU210 in rats subjected to permanent middle cerebral artery occlusion (PMCAO). They demonstrated that administration of HU-210 (45 μg/kg) at 1, 2, or 4 but not 6 hours after PMCAO resulted in reduced motor disability and infarct volumes compared with vehicle controls 72 hours after cerebral ischemia and thus, therapeutic time window appeared to extend to 4 hours after PMCAO and the salutary effects of HU-210 were only partially abolished by the CB_1_ receptor antagonist SR141716 and warming [18]. They concluded that the neuroprotective effects of HU-210 were dependent on potent hypothermia via CB_1_ receptor dependent mechanism. Another research group noted that 40 min before the induction of global cerebral ischemia, treatment with R(+)-WIN55,212-2 (1 mg/kg), a synthetic aminoalkylindole cannabinoid CB_1_ receptor agonist, induced a dose-dependent increase in neuronal survival that reached maximal levels (56% of sham-operated controls) in a global cerebral ischemia model in rats. Treatment with R(+)-WIN55,212-2 (1 mg/kg) 30 min before ischemia, but not 60–120 min after ischemia, reduced the infarct size 24 hours after ischemic stroke onset [72], suggesting that pre-ischemic treatment with R(+)-WIN55,212-2 is protective in cerebral ischemia.

### 3.3. A Synthetic Non-Competitive NMDA Antagonist, HU-211, Dexanabinol

Dexanabinol (HU-211) is a synthetic, nonpsychotropic cannabinoid [90]. It has been shown to act as a noncompetitive *N*-methyl-D-aspartate receptor antagonist [91], as well as having antioxidant [92], anti-inflammatory effects [93,94]. The therapeutic time window of nonpsychotropic cannabinoid, HU-211 in closed head injury was first reported by Mechoulam’s research group in 1993 [73]. HU-211 dissolved in middle-chain triglycerides (MCT) oil at a dose of 25 mg/kg was given intraperitoneally immediately and 1, 2, or 3 h after impact. The drug was found to be very effective in improving motor function recovery when given 2 h after the injury. In addition, Shohami and co-workers have shown the long-term effect of HU-211 on motor and memory functions after closed head injury in the rat [74]. HU-211 (5 mg/kg) was administered intravenously at 4 or 6 h after closed head injury. Cognitive functions were evaluated using the Morris water maze, with rats trained either before or after closed head injury. The data showed that HU-211 was a potent cerebroprotective agent, with a therapeutic window of about 4 h. Moreover, this compound HU-211 was also used for cerebral ischemia. Belayev and co-workers have reported that HU-211 (4 mg/kg, i.v.) significantly improved neurological deficits and reduced brain damage 60 min after forebrain ischemia in rats, but neuroprotection was no longer significant after 3 h [75]. Leker’s research group demonstrated that treatment with HU-211, dexanabinol (4.5 mg/kg) 1 h and 3 h but not 6 h after permanent middle cerebral artery occlusion in rats improved motor function and reduced infarct volume 24 h post ischemia [76].

## 4. Therapeutic Possibility of CBD

Cannabinoids may play a role in neuroprotection in disorders such as stroke, Parkinson’s disease, traumatic brain injury and epilepsy. These disorders may be caused by the generation of free radicals, reactive oxygen species, and pro-inflammatory cytokines. Emerging data suggest that natural- and synthetic-cannabinoids show a potent anti-oxidant activity and anti-inflammatory reaction in *in vivo* and *in vitro* studies. Among cannabis compounds, CBD may represent a very promising agent with the highest prospect for therapeutic use. An Israeli pharmaceutical company called Pharmos was conducting human clinical trials using a synthetic dextrocannabinid, dexanabinol, which has neuroprotective properties through inhibition of NMDA glutamate receptors, as well as anti-inflammatory and antioxidant activities like CBD, in patients with severe traumatic brain injury [80] and had been granted orphan drug status by the FDA [81]. However, in December 2004, Pharmos announced that no efficacy was observed as measured by the primary clinical outcome endopoint in Phase III TBI trials. After this announcement, SAPHIR and Pharmos investigators noted that there was a potential for selection bias that may creep into well-designed randomized clinical trials as a result of factors outside the control of investigators [82]. In 2003, a clinical trial investigating the protective properties of CBD in neurodegeneration was also performed by GW pharmaceuticals, with the results still pending.

A cannabis based-extract with approx 1:1 ratio of delta^9^-THC and CBD (Sativex) is marketed in Canada for the symptomatic relief of neuropathic pain in adults with multiple sclerosis. Health Canada has issued a Qualifying Notice that enabled full approval in early 2005. Three years ago, a randomized controlled trial of cannabis-based medicine (CBM) was performed in patients with multiple sclerosis in the UK. The results showed that CBM improved the symptoms of spasticity in multiple sclerosis and that delta^9^-THC plus CBD was better tolerated than delta^9^-THC as a single molecule [77]. More recently, a randomized placebo-controlled double blind clinical trial of Sativex was performed in patients with painful diabetic neuropathy. This trial assessing the efficacy of Sativex in patient with depression has shown it to be no more efficacious than placebo. Patients with depression had significantly greater baseline pain scores that improved regardless of intervention [78]. 

More recent clinical trials in 2010 showed that adjunctive treatment with cannabidiol was safe, well tolerated and effective in the treatment of psychosis in patients with Parkinson’s disease [83]. Unfortunately, there are currently no clinical studies to investigate the cerebroprotective effect of CBD after stroke.

## 5. Conclusions

In the last 10 years, it has been possible to demonstrate that CBD has the following unique therapeutic profile: 1) a cannabinoid receptor-independent mechanism, 2) long-lasting cerebro- protective effect after ischemic stroke, and lack of development of tolerance. Moreover, CBD has almost no side effects, including psychotropic activity. Preliminary studies highlight the fact that the multifunctional actions of CBD may lead to benefits in more complex systems within the brain after ischemic stroke. CBD offers new therapeutic possibilities for treating ischemic stroke, although further clinical studies are needed to definitively evaluate the clinical values of CBD. 
